# Bladder contracture associated with diffuse nephrogenic adenoma: a case report

**DOI:** 10.3389/fmed.2026.1936462

**Published:** 2026-09-09

**Authors:** Ning Xu, Qi Zhang, Xiangcheng Qin

**Affiliations:** Department of Urologic Surgery, Ningbo Hospital of Integrated Traditional Chinese and Western Medicine, Ningbo, Zhejiang, China

**Keywords:** bladder contracture, ileal bladder replacement, nephrogenic adenoma, pathology, tumor

## Abstract

A 52-year-old man presented with severe irritative lower urinary tract symptoms. Cystoscopy revealed extensive scattered papillary tumor-like lesions, mainly involving the lateral and posterior bladder walls. Computed tomography urography (CTU) showed bladder-wall thickening and reduced bladder capacity. Endoscopic biopsy and transurethral resection of bladder tumor (TURBT) were performed on two occasions; both histopathological examinations demonstrated suppurative inflammation, marked acute and chronic inflammatory cell infiltration, granulation-tissue proliferation, and focal lymphoid hyperplasia. Empirical antituberculous therapy was administered for suspected tuberculosis, but the irritative symptoms persisted. Repeat cystoscopy 6 months later again demonstrated extensive scattered papillary tumor-like lesions. Another TURBT was performed, and subsequent pathological consultation established the diagnosis of bladder nephrogenic adenoma. During follow-up, bladder capacity continued to decline, and retrograde cystography demonstrated left vesicoureteral reflux after instillation of only 30 mL of contrast medium. Because bladder contracture severely impaired quality of life, radical cystectomy with orthotopic ileal neobladder substitution was ultimately performed. At 3 months postoperatively, the voiding interval had increased to 1–2 h, nocturia had decreased to approximately three episodes per night, and the perineal radiating pain had resolved, without significant urgency or urinary incontinence.

## Introduction

Nephrogenic adenoma is a benign metaplastic lesion that occurs predominantly in adult men (1), and the bladder is its the most frequent site (1). Proposed pathogenic mechanisms include urothelial metaplasia and the detachment and implantation of renal tubular cells, usually following trauma or inflammatory stimulation (1).

Bladder contracture, is uncommon, and its causes, diagnosis, and treatment, remain insufficiently documented; tuberculosis is the most common cause (2). Ketamine and chemotherapeutic agents including doxorubicin and mitoxantrone, have been implicated in the development of bladder contracture (3–5). Other uncommon causes include eosinophilic cystitis and radiation-induced contracture (2). Here, a rare case of bladder contracture associated with diffuse nephrogenic adenoma in a male patient is reported.

## Case presentation

The 52-year-old male patient had initially been admitted to a local hospital at 49 years of age for “recurrent urinary frequency and urgency persisting for one year.” The average voiding interval was 20–40 min, and nocturia occurred more than 10 times per night. His medical history was unremarkable, with no hypertension, diabetes, other chronic disease, long-term medication use, or relevant family history. Histopathological examination of a transurethral bladder mucosal biopsy obtained at the local hospital showed suppurative inflammation. No prostatic hyperplasia or urethral abnormalities were identified during the endoscopy. After 1 year of treatment at the local hospital, the patient presented to our hospital. Multislice spiral computed tomography (CTU) demonstrated marked bladder-wall thickening and reduced bladder capacity (see Supplementary Figure S1). Both the urinary acid-fast bacillus smear and the urinary Mycobacterium tuberculosis DNA test were negative. Urinalysis showed a white blood cell count of 4,020/*μ*L, and urine culture yielded Candida glabrata.

Cystoscopy showed postoperative scarring changes, erosive changes involving the posterior bladder wall and trigone, dark-red edematous mucosa, and punctate bleeding. After antifungal treatment with itraconazole, transurethral resection of the bladder lesion was performed to establish the diagnosis further.

Postoperative histopathological examination revealed chronic inflammatory foci with suppurative inflammation, granulation-tissue hyperplasia, and dense infiltration by lymphocytes, neutrophils, and eosinophils. Small foci were suspected to represent necrosis. Antituberculous therapy with isoniazid, rifampicin, and ethambutol was administered for 3 months. During treatment, the voiding interval remained 5–8 min, accompanied by radiating pain in the penis and perineum.

**Figure 1:**
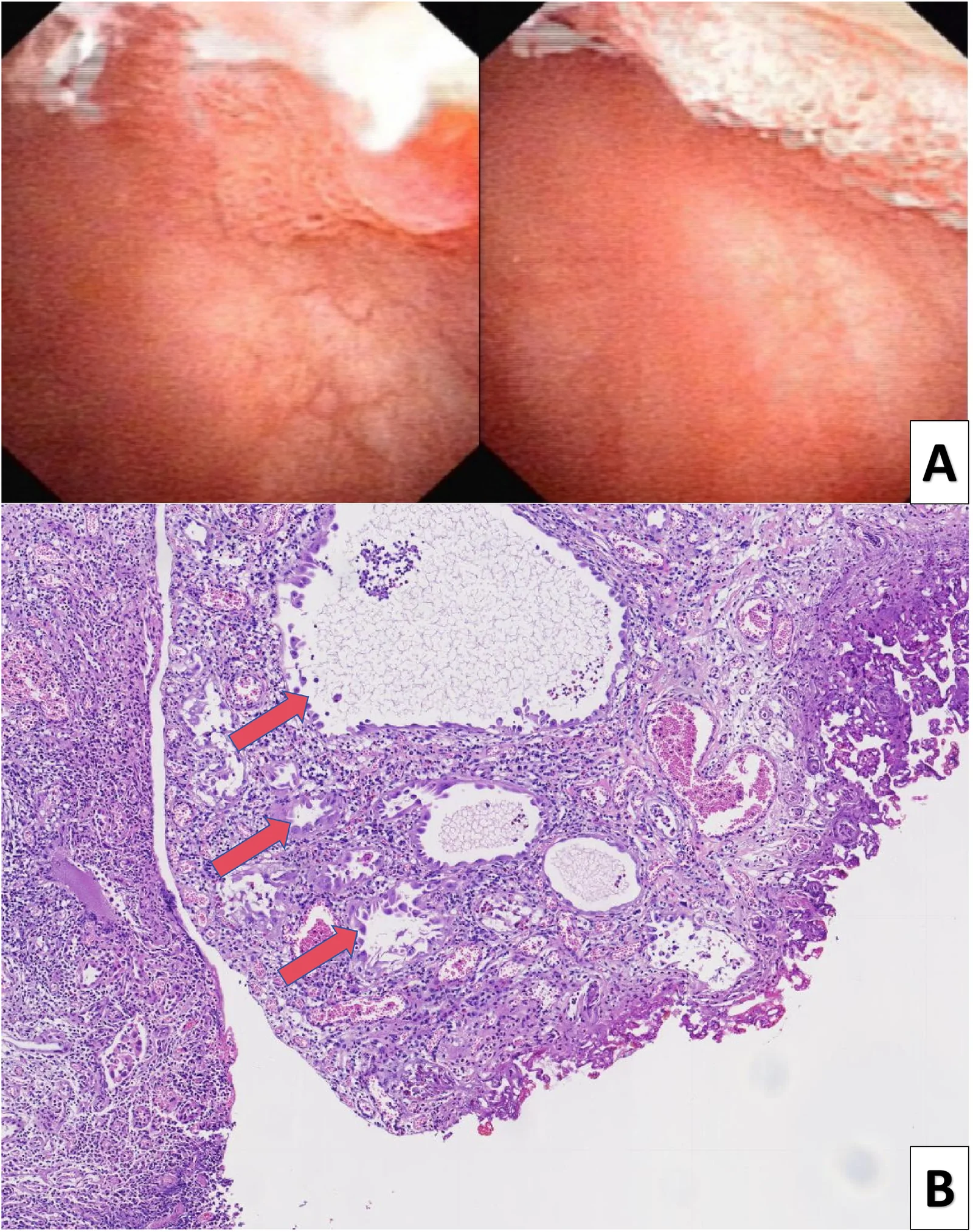
**(A)** Cystoscopic examination showing extensive papillary tumor-like lesions throughout the bladder **(B)** histopathological findings after surgery. Hematoxylin and eosin-stained sections showed chronic inflammation of the urinary tract mucosa with fibrous hyperplasia and multifocal distribution of tubules, microcysts, and isolated or small nests of epithelioid cells in the lamina propria. Hobnail cells were present, whereas signet-ring cell changes were rare. Interstitial mucin accumulation, edema, and chronic inflammatory-cell infiltration were also observed. No muscular layer involvement, no mitotic activity, no nuclear atypia and no stromal reaction were observed. The red arrows indicate, from top to bottom, microcysts, tubules, hobnail cells.

**Figure 2:**
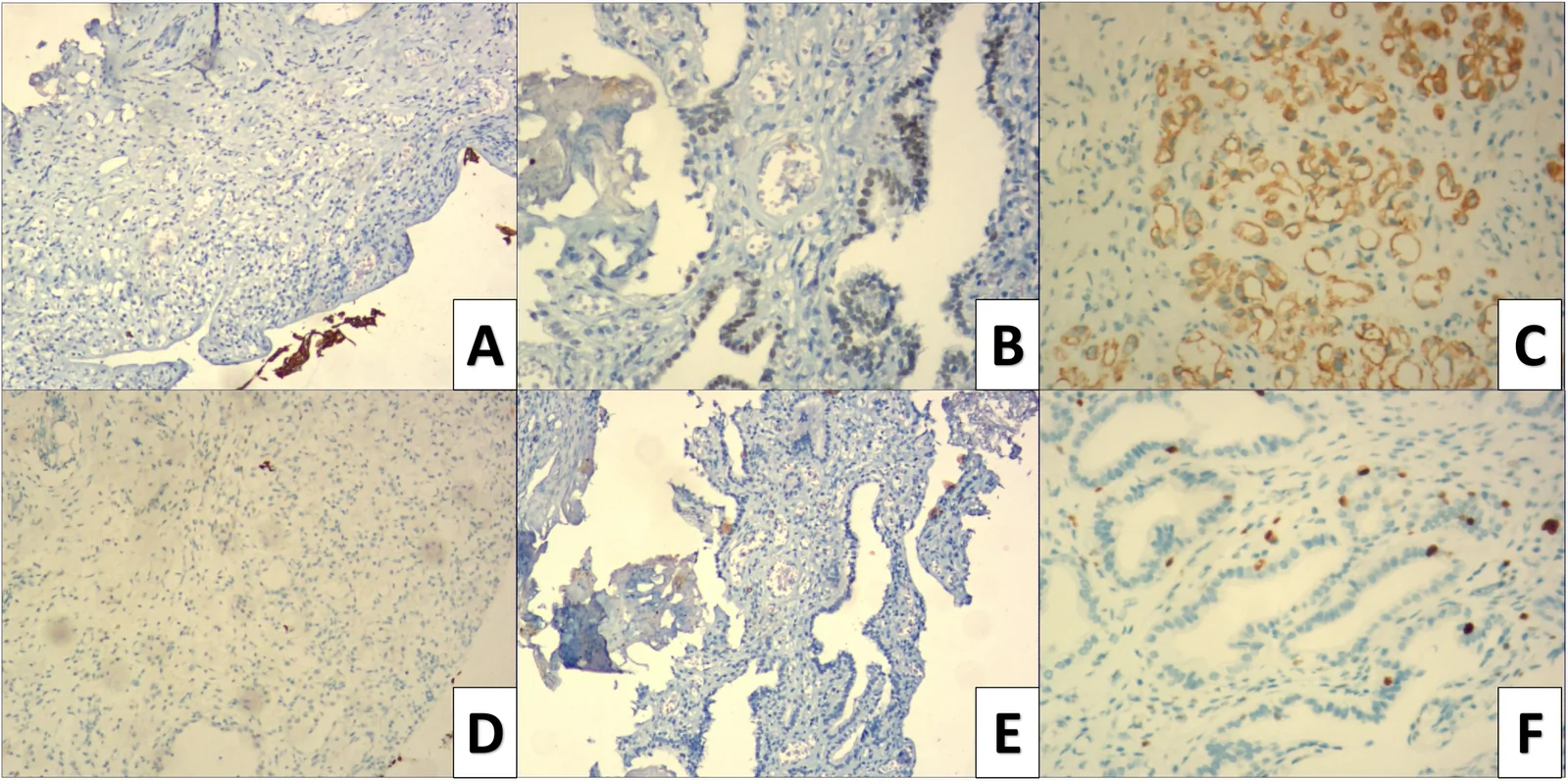
Immunohistochemical staining showed CK (pan) (+), GATA3 (−), CK20 (−), p63 (−), P504S (+), CEA (−), Ki-67 (+, 5%), PAX8 (+), napsin A (−), and CK34*β*E12 (+). **(A)** CK34*β*E12, 200X. **(B)** PAX8, 200X. **(C)** CK (pan), 200X. **(D)** p63, 100X. **(E)** Napsin A, 200X. **(F)** Ki-67 proliferation index was approximately 5%, 200X.

After 6 months of treatment at our hospital, repeat cystoscopy demonstrated extensive small papillary bladder lesions, resembling tumor-like changes (Figure 1A). The suspicious lesions were again resected by TURBT, and the specimens were submitted to a referral hospital for pathological consultation (Figure 1B). The immunohistochemical findings ultimately established a definitive diagnosis of nephrogenic adenoma (Figure 2). The patient was subsequently managed by clinical observation and follow-up.

**Figure 3:**
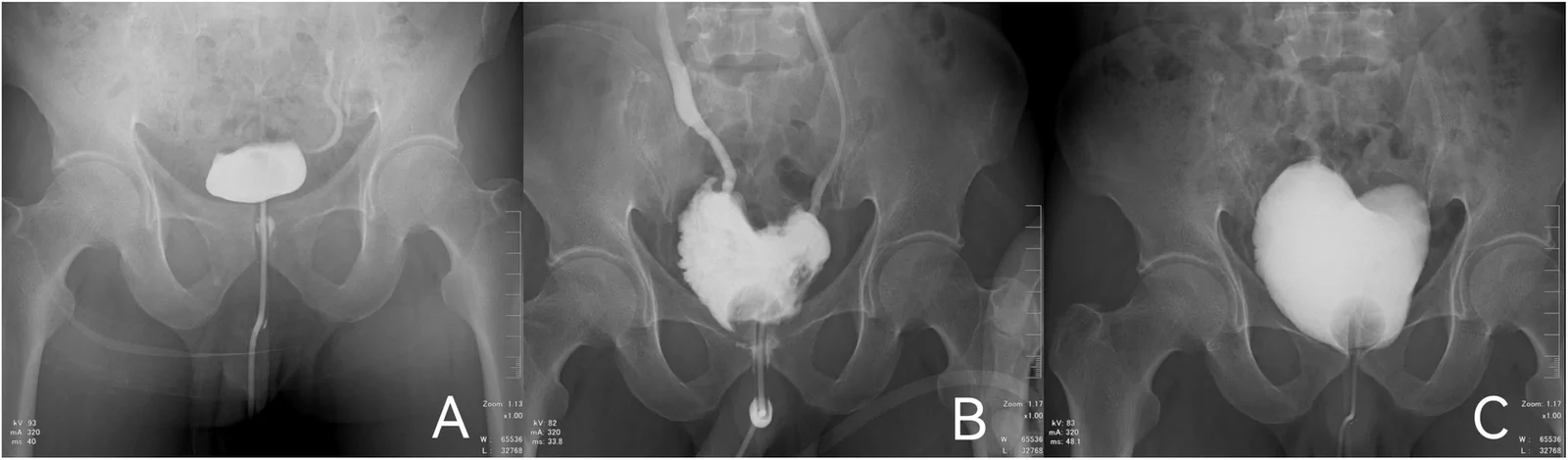
Cystographic findings. **(A)** Preoperative cystography showed markedly reduced bladder capacity and left vesicoureteral reflux after instillation of 30 mL of contrast medium. **(B)** At 15 days after surgery, bladder capacity had increased to 80 mL. **(C)** At 110 days after surgery, bladder capacity had increased to 250 mL.

Urinary frequency progressively worsened, with the voiding interval decreasing from 15 to 20 to 10–15 min. Therefore, cystography was performed during the second year of follow-up at our hospital (Figure 3A). Urodynamic testing showed an initial sensation at 17 mL, initial urgency at 24 mL, and a maximum bladder filling volume of 28 mL. The bladder contracture was more severe than that observed at the patient's presentation 2 years earlier. However, his renal function remained normal.

Persistent urinary frequency, urgency, and chronic pain markedly impaired the patient's psychological well-being and daily activities. The severely reduced bladder capacity made cystoscopy under local anesthesia intolerable, and the patient declined further TURBT. Because the potential effects of residual nephrogenic adenoma in the retained bladder after augmentation cystoplasty could not be excluded, the patient ultimately chose radical cystectomy with orthotopic neobladder substitution.

**Figure 4:**
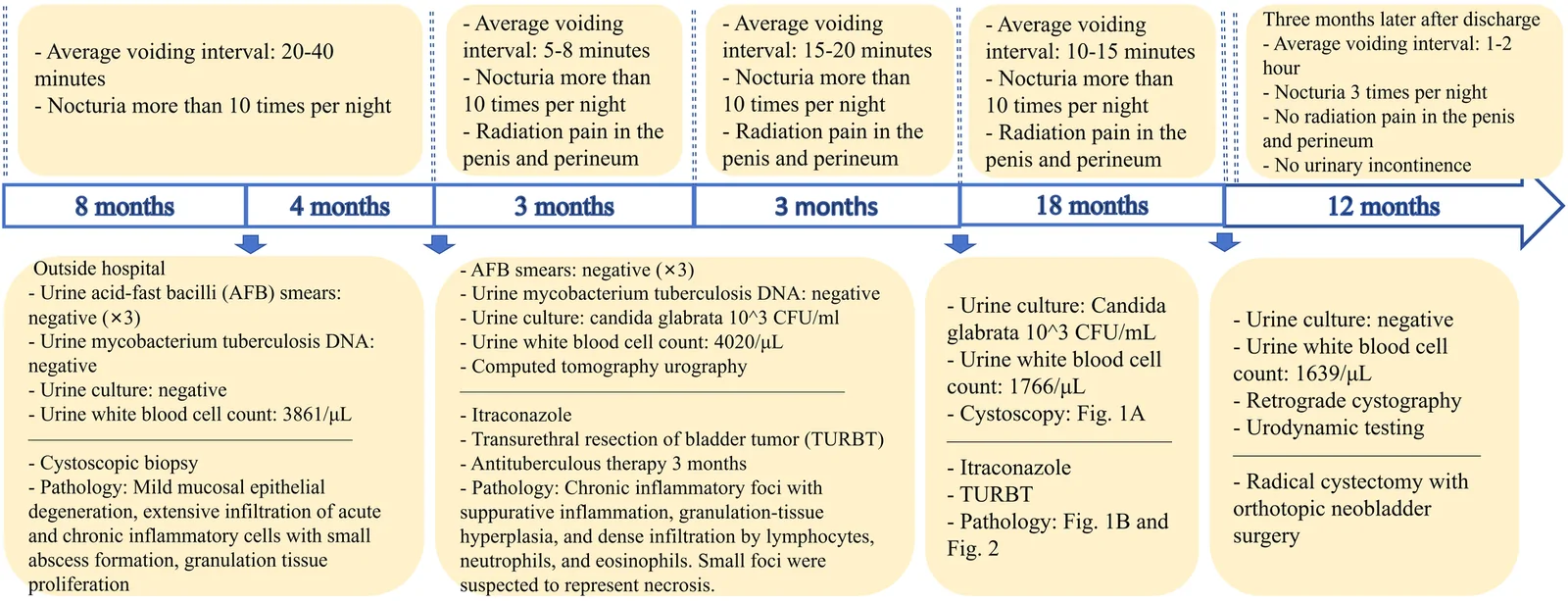
Timeline of the principal clinical events.

The patient was followed for 1 year after surgery. At 3 months after discharge, the voiding interval had increased to 1–2 h, and nocturia had decreased to approximately three episodes per night. No significant urgency or urinary incontinence was reported, and radiating pain in the penis and perineum had completely resolved. Renal function remained normal without evidence of hydronephrosis. Cystography was repeated 15 days (Figure 3B) and 110 days (Figure 3C) after surgery. The principal clinical events are summarized in the timeline shown in Figure 4.

## Discussion

Nephrogenic adenoma is a rare benign metaplastic lesion of urothelial origin (6). The bladder is the most commonly affected site (80%), followed by the urethra (15%) and ureter (5%). Previous reports have closely linked the development of nephrogenic adenoma to persistent urothelial irritation (7). Reported associated include urothelial carcinoma (38.7%), urolithiasis (29%), chronic urinary tract inflammation (19.4%), endoscopic procedures (83.9%), bladder surgery (3.2%), and urethral diverticulum (6.5%) (8).

In the present case, prominent irritative urinary symptoms, perineal radiating pain, microscopic hematuria, and pyuria were observed, consistent with previous reports (9). Despite the absence of relevant medical history, the patient was initially misdiagnosed with urinary tuberculosis because conventional anti-infective therapy was ineffective, the bladder wall was thickened, bladder capacity was reduced, and small chronic granulomatous inflammatory foci were considered suspicious for caseous necrosis. The inflammatory histopathological findings may also have been related to the preceding chronic urinary tract infection, which may have contributed to the development of nephrogenic adenoma in this patient. Moreover, chronic urinary tract infection in this patient suggested a potential inflammatory etiology for the bladder contracture (10). However, the rate and severity of the reduction in bladder capacity were not commensurate with the severity of the urinary tract infection. Therefore, this condition could not be simply attributed to inflammatory bladder contracture.

Microscopically, nephrogenic adenoma is characterized by proliferating tubules and microcysts surrounded by a thin rim of basement membrane-like hyaline material (11). Immunohistochemistry is highly valuable for diagnosing nephrogenic adenoma. The lesion is typically positive for P504S, pan-cytokeratin AE1/AE3, CK7, CAM5.2, epithelial membrane antigen (EMA), CD10, Napsin A, and high-molecular-weight cytokeratin 34*β*E12, with patchy staining for the latter (12). In diagnostically challenging cases, PAX8 positivity combined with the absence of p63 and GATA3 is often sufficient to support the diagnosis (13). Although Napsin A is considered a sensitive marker and is useful in the differential diagnosis of nephrogenic adenoma (14), staining was negative in the present patient. All reported fibromyxoid variants of nephrogenic adenoma have been negative for Napsin A, and approximately two-thirds have also contained a classic component, in which Napsin A immunoreactivity was retained (15). The fibromyxoid variant is characterized by spindle-cell proliferation within a fibromyxoid stroma (15). However, no fibromyxoid morphology was identified in this case, and the reason for the absent Napsin A expression remains unclear. The remaining immunohistochemical findings were consistent with those described previously.

Flat intravesical lesions in male patients may further complicate the diagnosis, but immunohistochemistry facilitates distinction from malignant mimics. Urothelial carcinoma is positive for GATA 3, p63, and uroplakin proteins but negative for PAX2 and PAX8 (16). Prostatic adenocarcinoma is negative for PAX8 but positive for PSA and NKX3.1 (17). Supplementary Table S1 summarizes the differential diagnosis of nephrogenic adenoma and other intravesical malignancies, including clear cell carcinoma (18, 19) and the microcystic variant of urothelial carcinoma (20).

Nephrogenic adenoma can also present as papillary, polypoid, exophytic, clear cell, signet ring-like or fibromyxoid patterns (9). Bladder lesions are generally small (<1 cm), although sizes up to 7 cm have been reported (21). Most lesions are solitary, approximately 20% are multifocal, and diffuse bladder involvement is rare (15, 21). Even in multifocal disease, TURBT remains the preferred approach for both diagnosis and treatment (8, 15). In the absence of concomitant malignancy, only two cases of total cystectomy for severe irritative bladder symptoms have been reported (22, 23). No previous report of augmentation cystoplasty for intravesical nephrogenic adenoma complicated by bladder contracture was identified.

Reported recurrence rates for nephrogenic adenoma vary among studies. One study documented recurrence after initial resection in only 14.7% of patients (24). Gordetsky et al. observed recurrence in 1 of 31 patients (8). By contrast, Zougkas et al. reported 1–7 recurrences in three of four patients (25). Previous TURBT has been identified as an the independent predictor of recurrence (26). No specific guidelines are available for follow-up of nephrogenic adenoma. Nevertheless, because recurrence or progression may occur, regular surveillance is recommended (24, 25). The rarity of this condition limited the available evidence for selecting treatment in the present case. Given the severe impairment of daily life and the bladder contracture objectively confirmed by urodynamics and cystography, radical surgery rather than repeated TURBT was considered justified.

## Conclusion

The clinical manifestations of nephrogenic adenoma are nonspecific, and most patients present with mild or poorly defined symptoms. Histologically, the lesion can be difficult to distinguish from primary or metastatic tumors of the urinary tract. In this case, nephrogenic adenoma showed a multicentric, infiltrative pattern with extensive involvement of the bladder mucosa. Rapid recurrence and progressive bladder contracture during diagnosis and treatment resulted in severe irritative urinary symptoms and radiating pain. Both the diffuse lesion burden and the intensity of symptoms were unusual. This case indicates that extensive recurrent nephrogenic adenoma can be associated with severe bladder contracture.

## Data Availability

The original contributions presented in the study are included in the article/Supplementary Material, further inquiries can be directed to the corresponding author.
