# Supplementary material for: Bladder contracture associated with diffuse nephrogenic adenoma: a case report
Source: Front Med (Lausanne). 2026 Sep 9;13:1936462. doi: 10.3389/fmed.2026.1936462 (PMC13597395; doi:10.3389/fmed.2026.1936462)
Supplement: Supplementary file 1 [file Table1.docx]

**Figure S1****.** CTU images: (A) The arterial phase (B) excretory phase.

| **Table 1** Nephrogenic Adenoma and Major Differential Diagnostic Considerations: Immunohistochemistry | | | | | |
| --- | --- | --- | --- | --- | --- |
|  | Nephrogenic Adenoma | Microcystic variant urothelial carcinoma | Clear cell carcinoma | Prostatic adenocarcinoma | Urothelial carcinoma |
| Immunohistochemistry | Positive PAX8, 34βE12, Ki-67(0-5%);  Negative p63, PSA, CEA, GATA3 | Positive CK7, CK20, p63, GATA3, MUC1 | Positive CK, CK7, CK20, CEA, Ki-67(10-80%);  Negative CD10 | Positive PSA, PSAP, NKX3.1;  Negative PAX8 | Positive GATA3, p63;  Negative PAX2, PAX8 |
